# A crowdsourcing open platform for literature curation in UniProt

**DOI:** 10.1371/journal.pbio.3001464

**Published:** 2021-12-06

**Authors:** Yuqi Wang, Qinghua Wang, Hongzhan Huang, Wei Huang, Yongxing Chen, Peter B. McGarvey, Cathy H. Wu, Cecilia N. Arighi, on behalf of the UniProt Consortium

**Affiliations:** 1 Protein Information Resource, University of Delaware, Newark, Delaware, United States of America; 2 Center for Bioinformatics and Computational Biology, University of Delaware, Newark, Delaware, United States of America; 3 College of Agriculture and Natural Resource, University of Delaware, Newark, Delaware, United States of America; 4 Protein Information Resource, Georgetown University Medical Center, District of Columbia, United States of America

## Abstract

The UniProt Knowledgebase is a public database for protein sequence and function, covering the tree of life. This Community Page article present a community submission system to harness timely scientific knowledge via crowdsourcing of the literature, creating a research ecosystem where researchers play an active role in scaling up UniProt curation, while receiving proper attribution for their biocuration work.

## Teaser

The UniProt knowledgebase is a public database for protein sequence and function, covering the tree of life and over 220 million protein entries. Now, the whole community can use a new crowdsourcing annotation system to help scale up UniProt curation and receive proper attribution for their biocuration work.

UniProt is a public source of protein sequence and function information. It includes proteins from the entire tree of life, serving a wide user community across diverse fields. The UniProt knowledgebase (UniProtKB) contains protein information derived by expert curation (Swiss-Prot section) and automated annotation (TrEMBL section) [1]. Expert curation involves a critical review of the experimental data from the literature and interpretation of the results from sequence analysis tools [2]. Currently, the UniProtKB contains over 220 million protein entries (Release 2021_03). Proteins from most organisms are unreviewed and only annotated via automatic annotation. This is due in part to a lack of direct experimental data available and a limited curation task force. Still, there is a significant number of proteins with some level of characterization or new information, awaiting annotation from the literature. Many UniProt users are highly engaged and motivated in providing feedback about entries in the database, especially when data are missing or an update is needed as UniProtKB is a highly valued resource and critical to their work. Users have vested interest that the knowledgebase is comprehensively annotated.

Engaging the research community in helping support curation or directly curating a resource has been an ongoing and evolving effort by many biological knowledge resources [3–5]. Here, we present a novel crowdsourcing community annotation system that enables researchers to quickly add annotations from the literature to UniProtKB protein entries. As opposed to past feedback options, this mechanism provides a systematic solution for community input, with a fast turnaround for presenting the added information to the community, and uses ORCID (https://orcid.org/) for authentication and acknowledgment of the contributions.

There are many reasons to contribute. As an expert on a given subject area, you are up-to-date with the literature and know what information is missing for a protein of interest. Thus, you can play an important and active role in improving the database and helping scale up curation. An improved database better supports the entire research ecosystem and moves science forward. To link a publication to a UniProt entry, you do not have to be its author, but to understand the content. In return, you get recognition for the biocuration work you do, you can cite it, and add it to your research portfolio.

## The community submission system

Our lightweight solution allows UniProt users to add publications and annotations to proteins of their interest, with a simple design and requiring minimal information, while providing options to contribute more details, and, most importantly, for acknowledging contributions. To develop such system, we adopted a user-centric approach, where a prototype was released to the community and feedback was collected through a survey to improve the design. The project homepage can be accessed at https://community.uniprot.org/bbsub/home.html and offers links to the submission system, the statistics, documentation, and code.

**Fig 1:**
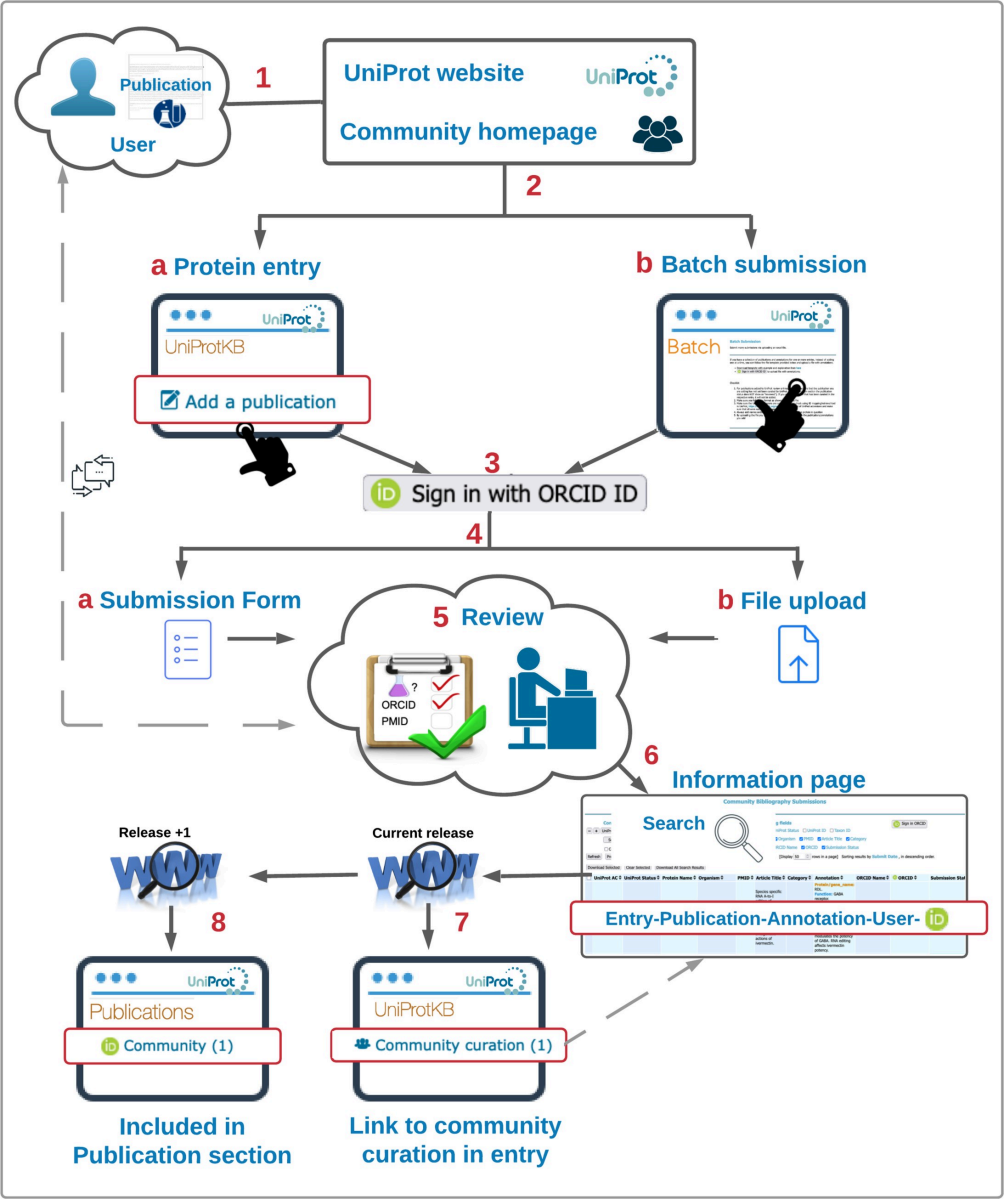
Overview of the submission process.

The submission process, described in Fig 1, starts with users and publication(s) to be added to one or more protein records. There are 2 access points, from the UniProt website or the community homepage (Fig 1, 1). To add one publication to a protein at a time, the “Add a publication” link in a UniProtKB entry page brings users to the submission page for such entry (Fig 1, 2a). Alternatively, a batch submission option is available from the homepage (https://community.uniprot.org/bbsub/batch.html) (Fig 1, 2b). ORCID identifiers are used for log in and contributor validation, as well as for credit attribution for community curation (Fig 1, 3). In the protein-centric submission case, the next step is to add the publication. The user has options to perform additional tasks, such as categorizing the publication into UniProt annotation topics and to input free text annotations related to protein/gene name, function, and disease (see sample form: https://community.uniprot.org/bbsub/sampleform.html) (Fig 1, 4a), whereas for submission in batch, the user would upload a file with multiple proteins, publications, and annotations (Fig 1, 4b). In both cases, submission triggers the review process (Fig 1, 5). Upon the reviewer’s approval, publications and annotations are publicly available on the submissions’ information page with ORCID and submitter’s name (https://community.uniprot.org/bbsub/bbsubinfo.html; Fig 1, 6) and will appear in the UniProt protein page under the “Community curation” link 24 to 48 h later (Fig 1, 7). Publications are also integrated into the UniProt bibliography in sync with the database release cycle (Fig 1, 8).

There are multiple checkpoints in place both to help the user and for data integrity, including (a) automatic retrieval of publication title and authors via PubMed API, to confirm the publication of interest; (b) checking that the publication has not been already curated by UniProt for such protein; (c) checking data changes in the upcoming UniProt release (private), like obsoletion or merging of entries, and warning the user to choose an alternative entry; (d) checking submissions are updates versus a new annotation; and (e) helping with text editing with auto-correction capability, warnings for non-ASCII characters, and checking for valid email format. In terms of information content, a UniProt curator ensures that the publication is associated to the correct protein entry (organism and accession number) and that the information added is concise and appropriate. If any problem arises, the curator contacts the user for clarification via email.

## Community participation and impact

The number of users and submissions have increased steadily since the inception of the system (UniProt Release 2019_08) (https://community.uniprot.org/bbsub/STATS.html). As of release 2021_03, a little more than half of the submissions provide annotations for unreviewed entries, whereas the rest offer updates for reviewed ones; roughly half of the contributions are done by students or researchers who are not authors on the submitted publications. Most importantly, the submissions add information to proteins from all superkingdoms and cover a variety of annotations topics, with majority related to Function, which is of high interest to UniProt. Regarding this last point, the community curation adds value to the UniProt entries. For example, it adds proper names to proteins annotated as uncharacterized in UniProt. Many offer functional information that is incorporated when publications are later curated by UniProt. For example, in release 2020_06, UniProtKB:Q8DPQ3 was an unreviewed entry with name “Uncharacterized protein” and lacking functional information (https://www.uniprot.org/uniprot/Q8DPQ3.txt?version=95); however, at the time, a community submitted publication added names (Pyrimidine nucleotidase A; PynA) and functional information to this entry (https://community.uniprot.org/bbsub/bbsubinfo.html?accession=Q8DPQ3). This publication and similar annotations were later incorporated when the entry was curated by UniProt (https://www.uniprot.org/uniprot/Q8DPQ3.txt?version=96). This and other representative examples (https://community.uniprot.org/bbsub/doc/public/uncharacterized_proteins.xlsx) show that the publications and annotations added by the community are relevant. The crowdsourcing effort also provides a number of annotations for unreviewed proteins, which can be retrieved in the information submission page, by selecting field “UniProt status” and “TrEMBL” in query box. In addition, the community submission system has enabled successful collaborations with research groups for improving annotations in defined research areas. **Box 1** describes 2 of these examples.

## Conclusions

Our system provides a solution for the scientific community to contribute their knowledge and enrich information for proteins in UniProt. Crowdsourcing the literature curation has been an asset both to UniProt and the users of the knowledgebase. Moreover, to cite your biocuration work, you can simply link to your public contributions using: https://community.uniprot.org/bbsub/bbsubinfo.html?ORCID=<ORCID>, where <ORCID> should be replaced with your ORCID.

**Box 1. Collaborations—Success stories**

Here are 2 illustrative examples of users who are actively providing valuable annotations to unreviewed entries for specific taxonomic groups.

1. **Annotation of *Giardia intestinalis* protein.** *G*. *intestinalis* is a eukaryotic parasite that causes giardiasis, with diarrhea and stomach cramps as main symptoms (https://www.cdc.gov/parasites/giardia/index.html). A search in UniProt (release 2021_03) with the query taxonomy “Giardia [5740]” retrieved 43,668 and 66 unreviewed and reviewed *Giardia* protein entries, respectively. A search in PubMed (accessed on June 30, 2021) with query terms “giardia AND (intestinalis or lamblia or duodenalis) AND “Amino Acids, Peptides, and Proteins”[MESH]” retrieved 1,825 articles, suggesting that a significant number of potentially relevant publications could be added to *Giardia* protein entries. Thus, Dr. Touz (Instituto Ferreyra, Cordoba, Argentina), an expert on *Giardia*, has helped in this task by contributing annotations and contacting other experts in the community. So far, over 50 unreviewed entries now have publications and annotations.
2. **Annotation of Haloarchaeal proteins.** Halophilic archaea are unique microorganisms adapted to survive under high salt conditions, and biomolecules produced by them may possess unusual properties, thus they are of high interest in the biotechnology field [6]. Dr. Pfeiffer (Max Planck Institute, Germany) has actively provided feedback to UniProt on the annotation of prokaryotic proteins. Since the community annotation functionality started, he has now contributed over 250 submissions, especially adding publications and annotation to proteins from haloarchaeal genomes while developing a strategy for curation of their corresponding genomes [7,8].
